# Unveiling ocular developmental disorders through short-read whole-genome sequencing

**DOI:** 10.1038/s41431-026-02160-4

**Published:** 2026-07-14

**Authors:** AURAGEN consortium, Bertrand Chesneau, Timotéo Cousteix, Abdelhakim Bouazzaoui, Hélène Dollfus, Marjolaine Willems, Audrey Putoux, Philippe Khau Van Kien, Vincent Michaud, Aurore Garde, Isabelle Meunier, David Geneviève, Elise Brischoux-Boucher, Fanny Laffargue, Justine Wourms, Marine Lebrun, Valentin Ruault, Julie Plaisancié, Nicolas Chassaing

**Affiliations:** 1 GCS AURAGEN, 69003 Lyon, France; 2 Laboratoire de Référence (LBMR) des anomalies malformatives de l’œil, Institut Fédératif de Biologie (IFB), CHU de Toulouse, Toulouse, France; 3 Centre de Référence des Affections Rares en Génétique Ophtalmologique, CARGO, CHU de Toulouse, Toulouse, France; 4 CNRS UMR5077, Centre de Biologie Intégrative, Université de Toulouse, Toulouse, France; 5 Centre Hospitalier Universitaire de Rennes, Service de Génétique Clinique, Centre de Référence Maladies Rares CLAD-Ouest, ERN ITHACA, Hôpital Sud, Rennes, France; 6 Centre de Référence Pour les Affections Rares en Génétique Ophtalmologique (CARGO), Institut de Génétique Médicale d’Alsace (IGMA), Filière SENSGENE, Hôpitaux Universitaires de Strasbourg, 67091 Strasbourg, France; 7 Département de Génétique Médicale, Maladies Rares et Médecine Personnalisée, Université de Montpellier, CHU de Montpellier, CLAD ASOOR Montpellier, Montpellier, France; 8 Hospices Civils de Lyon, Service de Génétique, Centre de compétence des affections rares en génétique ophtalmologique, Lyon, France; 9 Centre de Recherche en Neurosciences de Lyon, équipe GENDEV, INSERM U1028 CNRS UMR5292, Université Claude Bernard Lyon 1, Lyon, France; 10 UF de Génétique Médicale et Cytogénétique, Centre Hospitalier Régional Universitaire de Nîmes, Nîmes, France; 11 Service de Médecine Génétique, Hôpital Universitaire de Genève, CH1206 Genève, Switzerland; 12 Service de Génétique Médicale, CHU de Bordeaux, Bordeaux, France; 13 Centre de Référence Anomalies du Développement et Syndromes Malformatifs, FHU TRANSLAD, Centre Hospitalier Universitaire Dijon, Dijon, France; 14 MAOLYA, CHU Montpellier, Montpellier, France; 15 Centre de génétique humaine, CHU Besançon, Université Marie et Louis Pasteur, Besançon, France; 16 Service de Génétique Clinique, CHU Clermont Ferrand, Clermont Ferrand, France; 17 Service de génétique clinique, CHRU de Nancy, Nancy, France; 18 Département de Génétique, Centre Hospitalier Universitaire de Saint-Etienne, Saint-Etienne, France

**Keywords:** Medical genetics, Development, Genetic testing

## Abstract

Congenital eye malformations represent a clinically and genetically heterogeneous group of disorders. Despite advances in genetic testing, fewer than half of affected patients receive a definitive molecular diagnosis. However, obtaining a molecular diagnosis is crucial for these patients and their families. Whole genome sequencing (WGS) is now standard practice in the genetic investigation of patients, but its contribution has not been extensively evaluated in patients with ocular malformations. In this work, we performed short-read WGS in a cohort of 100 families presenting with eye developmental disorders, including microphthalmia-anophthalmia spectrum (M/A), coloboma, anterior segment dysgenesis, congenital cataracts and/or foveal hypoplasia. Prior to WGS, 47 individuals had undergone targeted next-generation sequencing (NGS) of genes panels related to ocular development, 11 had chromosomal microarray analysis (CGH-array) and 20 had a combination of both. None had received a definitive genetic diagnosis. WGS identified a (likely) pathogenic variant in eighteen patients. In addition, candidate variants of uncertain significance were detected in nine patients. Notably, fourteen of these variants would have been missed by conventional genes panels or CGH-array, underscoring the broader diagnostic scope of WGS. Our findings demonstrate that short-read WGS significantly improves diagnostic yield in patients with congenital eye malformations, including those previously undiagnosed despite NGS panel testing. These results support the integration of WGS in the genetic evaluation of ocular developmental disorders.

## Introduction

Structural eye disease encompasses a wide spectrum of structural anomalies resulting from abnormal eye development. It account for 10% of visual impairment observed in children, with a prevalence ranging from 3.5 to 4.8 cases per 10,000 living births [1, 2]. It includes ocular globe growth defects (M/A) form a spectrum that ranges from microphthalmia (small eye) of variable severity to anophthalmia (complete absence of the ocular globe) [3]. Ocular colobomas result from defects in optic fissure closure and are frequently associated with microphthalmia [2]. Congenital disorders resulting from abnormal development of the anterior segment of the eye (i.e., cornea, iris, trabecular meshwork, ciliary body and lens), also are frequent malformations of the eye, generally grouped as anterior segment dysgenesis (ASD) [4, 5]. Congenital cataracts are defined as loss of lens transparency diagnosed in the first year of life [6]. Patients may also exhibit abnormalities in the development of the posterior segment of the eye. These can include foveolar hypoplasia, which can be isolated or associated with albinism [7]. Although multiple causes have been identified to date, genetic causes remain predominant in ocular malformations [3, 8]. The ocular malformation can affect one or both eyes. It can be simple but association of multiple ocular defects is common (i.e. microphthalmia and ASD) resulting in a complex ocular malformation. Various extra-ocular features can also be found in 30 to 90% of individuals with M/A [3]. Individuals with other ocular defect (ASD, congenital cataract) may also exhibit non-ocular findings [9].

There is a genetic heterogeneity among these ocular development defects, with 150 genes associated with structural eye disease in the literature (PanelApp v5.0) [10]. Moreover, the involvement of several genes in multiple ocular and extra-ocular phenotypes further complicates phenotype-genotype correlations [3, 11]. In recent years, exome and genome sequencing (WES, WGS) have emerged as interesting tools to screen individuals with congenital eye anomalies [12–14]. This genomic screening has facilitated the description of new genes associated with eye development anomalies [15]. WGS also covers non-coding regions where a growing part of variants are described [16]. Moreover, it allows the detection of balanced structural variants (SVs). Despite these new sequencing approaches, less than half of individuals affected with ocular malformations have a genetic diagnosis after exploration. This number varies greatly between cohorts reported in the literature, depending on the phenotype and genetic analyses performed (Table 1). For M/A, 16-61% of individuals have a genetic diagnosis [17–23]. This is the same regarding ASD with 13-54% of affected individuals with a molecular diagnosis [4, 21, 23, 24, 25], when excluding aniridia which affects the whole ocular globe and is caused by a *PAX6* loss of function variant in more than 90% of cases [26]. Finally, a genetic diagnosis is found in 37-77% of individuals with congenital cataracts [21, 27–35].

**Table 1:** Cohort of individuals with ocular development anomalies genetically explored.

Study	Number of individuals	Population	Ocular phenotype	Individuals with extra-ocular features	Analyses performed before inclusion	Genetic analyses performed	Number of diagnoses (%)
Reis et al. 2013 [29]	18	United States	Familial congenital cataract	NA	None	WES (in silico panel of 36 genes)	11 (61%)
Chassaing et al. 2014 [36]	150	France	M/A	43%	None	Sanger sequencing: *GDF6, FOXE3, OTX2, PAX6, RAX, SOX2, VSX2*. QMPSF: *OTX2, RAX, SOX2, VSX2*.	32 (21%)
Matias-Perez et al. 2018 [17]	14	Mexico	M/A	50%	Karyotype (if severe M/A and ID)	WES	8 (60%)
Patel et al. 2018 [18]	M/A 66 Posterior micro 23	Saudi Arabia ("highly consanguineous population")	M/A	37%	SNP-array, karyotype	WES, multigene panel (322 genes)	M/A (61%) Posterior microphthalmos (82%)
Jackson et al. 2020 [21]	293	United Kingdom (White British 25%, White other 15%, Asian Pakistani 7%, Asian Bangladeshi 4%, Asian Indian 4%, Asian other 8%, Black African 3%, Black Caribbean 1%, Black other 3%, mixed White/Black African 3%, other 15%, NA 11%)	M/A (70) ASD (46) Congenital cataract (38) Other (139)	NA	NA	short-read WGS	Overall 73 (24.9%) *M/A 11 (15.7%)* *ASD 6 (13.0%)* *Cataract 17 (44.7%)*
Ma et al. 2020 [24]	41	Australia	ASD	29%	NA	CGH-array,WES, short-read WGS	22 (54%)
Islam et al. 2020 [20]	19	Pakistan ("population with high rates of consanguinity")	M/A	26%	NA	SNP arrays, WES	1 (5%)
Ma et al. 2021 [27]	52	Australia	Congenital cataract (52)	0%	CGH-array and/or targeted NGS-WES (14/52)	short-read WGS	40 (77%) *including 5 (10%) only detectable with WGS*
Haug et al. 2021 [19]	19 (15 families)	Switzerland	M/A, Coloboma	32%	NA	WES	7 (47%)
Li et al. 2022 [12]	67	United States of America (Non-Hispanic White 61%, Hispanic 28%, Non-Hispanic Black 5%)	M/A	18%	Exclusion of individuals with a recognizable phenotype or suspected to have a chromosomal anomaly	WES trio	14 (20.9%)
Chesneau et al. 2022 [25]	95	France	ASD (Peters' anomaly)	41%	NA	NGS panel (119 genes) +/− CGH-array	34 (36%)
Harding et al. 2023 [22]	50	United Kingdom	M/A (17/50 syndromic)	34%	NA	CGH-array +/− targeted NGS or short-read WGS	15 (33%) *including 4/17 (24%) for cases who were analyzed with WGS*
Kunisetty et al. 2024 [13]	189	United States of America	M/A (syndromic)	100%	CGH-array	WES	61 (32.3%)
Zucco et al. 2024 [4]	162	Italy	Aniridia (129) ASD (8) M/A (22) Morning glory (3)	NA	NA	WES +/− targeted PAX6 sequencing/MLPA	Overall 125 (77.2%) *Aniridia (89.9%)*
Wang et al. 2024 [28]	115	China	Congenital cataract	40%	NA	WES	(72.2%)
Lecca et al. 2024 [30]	67 (51 families)	Italy	Congenital cataract	25%	None	WES	19 (37%)
Atique Tacla et al. 2024 [14]	17	Brazil	M/A	100% (orofacial clefts)	NA	Microarray, WES	6 (35.29%)
Neuhann et al. 2025 [23]	40	Germany	M/A (22) ASD (14) Congenital cataract (4)	28%	None	primary diagnostic testing, multigene panel (186 genes), WES	17 (43%) *M/A 8 (36%)* *ASD 6 (43%)* *Congenital cataract 3 (75%)*
Zhang et al. 2025 [31]	114	China	Congenital cataract	9%	NA	WES	49 (43%)
Guo et al. 2025 [33]	150	China	Congenital cataract	NA	NA	NGS panel (39 genes)	95 (63%)
Jones et al. 2025 [34]	20	Myanmar	Paediatric cataract (36% associated with M/A)	NA	None	WES	9 (45%)
Sun et al. 2025 [35]	58 (19 families)	China	Congenital cataract	NA	None	WES	8/19 families (42%)
Huang et al. 2026 [32]	40	Southern China	Congenital cataract	20%	NA	WES	15 (37,5%)
**This study**	100	France	M/A +/− ASD (54) ASD (20) Coloboma (16) Congenital cataract (6) FH (4)	55%	CGH-array (n = 31), targeted NGS (n = 67)	shord-read WGS	Overall 18 (18%) *M/A +/− ASD 10 (19%)* *ASD 4 (22%)* *Coloboma 2 (13%)* *Cataract 2 (33%)* *FH 1 (25%)*

ASD: Anterior Segment Dysgenesis, M/A: Microphthalmia-Anophthalmia, MLPA: Multiplex Ligation-dependent Probe Amplification, NGS: Next Generation Sequencing, QMPSF: Quantitative Multiplex Polymerase Chain Reaction (PCR) amplification of Short fluorescent Fragments, WES: Whole Exome Sequencing, WGS: Whole Genome Sequencing.

To date, only four cohorts of individuals with structural eye disease explored using WGS have been reported [21, 22, 27]. Furthermore, the diagnostic rates for individuals with M/A (15.7% and 33%) are similar to that obtained through targeted sequencing of the main genes involved in these anomalies (21%) [36]. This work aims to evaluate the diagnostic yield and define the genetic spectrum of a diverse cohort of 100 unrelated individuals with structural eye diseases using WGS. Specifically, we aim to assess the added value of WGS in detecting complex structural variants and exploring non-coding regions, comparing our findings to established diagnostic rates in the literature to determine its utility as a first- or second-tier diagnostic tool.

## Material and methods

### Patients

This study was designed in compliance with the Helsinki Declaration and informed consent was obtained from the patient or their legal guardians. All included individuals were explored as part of the French Genomic Medicine Initiative (PFMG2025) [16] with “Eye malformation” as indication for the analysis.

Two-thirds of individuals (67/100) had previously benefited from NGS analysis, either a targeted panel of 24 (*n* = 8), 119 (*n *= 36) or 187 (*n* = 13) genes associated with eye development anomalies (solo analyses, performed in Toulouse University Hospital, the lists of genes are available in Supplementary Data 1), another panel of genes (*n* = 7) or WES (*n* = 3, trio analyses) (details in Supplementary Data 2). Furthermore, 31 individuals underwent CGH-array: 20 of these had also received NGS analysis, while 11 had no prior NGS testing. These analyses did not identify any causative variant.

Of note, four individuals (I010, I023, I030 and I062) have been previously reported as part of other cohorts [37–39].

### Genetic analysis

WGS was performed by Auragen consortium following the recommendations of PFMG2025 [16]. In addition to the index case, relatives were sequenced in 99% of cases to facilitate the interpretation of genomic findings within the family. Family structures consisted of 79 trios (index and asymptomatic parents), 12 trios with additional symptomatic relatives, 2 quartets, 2 duos with an asymptomatic parent, 1 duo with a symptomatic relative, 1 solo analysis and 3 involving other relative combinations. Whole blood extracted genomic DNA was sequenced according to standard procedures for a PCR-Free genome on a NovaSeq6000 instrument (Illumina – San Diego, California, United States). Sequencing data were aligned to the GRCh38p13 full assembly using bwa 0.7 + . Variants were called by several algorithms including GATK4 + , Bcftools1.10 + , Manta1.6 + , CNVnator0.4 + , and annotated using the variant effect predictor. Variant selection focused on the index case, with relative data used to guide the diagnostic interpretation. ClinVar-labeled pathogenic variants were systematically prioritized. Inclusion criteria required >2 reads, an allelic balance ≥10%, and rarity in population databases ( < 1% in gnomAD and <10% in the internal Auragen asymptomatic cohort). Additionally, variants were restricted to those located within 2 kb of UTR boundaries. Subsequent prioritization was performed via in-house procedures. Briefly, it integrates segregation, variant impact, and phenotypic relevance (based on clinician-provided HPO terms using Phrank score [40]). Notably, Single Nucleotide Variants (SNVs) and insertions/deletions (indels) predicted to alter the splicing of genes of interest were prioritized using SpliceAI ( ≥ 0.2) and/or CADD (v1.6 phred > 25). Finally, non-prioritized variants underwent manual expert review via SeqOne, incorporating clinical context and geneticist expertise. Further details are available on request.

Given that all reported SNVs and indels exhibited a sequencing depth above 30X and an allele frequency of at least 40%, further validation by an alternative method was not required. Reported inversions (inv(6p25.1p25.3), inv(10q24.3q26.1), inv(11p11.2p13)) were confirmed by Sanger sequencing of breakpoints of genomic DNA extracted from a second blood sample.

## Results

We report here a cohort of 100 unrelated individuals affected with eye development anomalies. Around half of them exhibit an eye malformation belonging to the M/A spectrum (*n* = 54/100) (Table 2, details available in Supplementary Data 2). Half of individuals with M/A (*n* = 27) had an eye malformation that was accompanied by an ASD phenotype. The remaining individuals had isolated ASD without ocular growth defect (*n* = 20), coloboma (*n* = 16), congenital cataract (*n* = 6) or foveal hypoplasia (*n* = 4). More than half of them also exhibit systemic features (*n* = 55/100), notably neurodevelopmental disorders (*n* = 19; namely: developmental delay (DD), intellectual development disorder (ID), autism spectrum disorder or learning difficulties), cerebral MRI anomalies (*n* = 7) and congenital genitourinary anomalies (*n* = 6) (Supplementary Data 2). Furthermore, 18 probands had at least one relative affected with a congenital eye malformation in a multigenerational family history.

**Table 2:** Summary of WGS results according to patient’s phenotypes.

	Number of individuals				
	Total	Conclusive	VUS	Partially conclusive	Inconclusive
Overall	100	18 (18%)	9 (9%)	6 (6%)	67 (67%)
**Gender**					
Female	43	8 (19%)	3 (7%)	4 (9%)	28 (65%)
Male	57	10 (18%)	6 (11%)	2 (4%)	39 (68%)
**Ocular phenotype**					
M/A	16	2 (13%)	4 (25%)	0	10 (63%)
M/A + Coloboma	11	2 (18%)	1 (9%)	0	8 (73%)
Coloboma	16	1 (6%)	1 (6%)	3 (19%)	11 (69%)
M/A + ASD	27	6 (22%)	1 (4%)	2 (7%)	18 (67%)
ASD	20	4 (20%)	0	0	16 (80%)
Cataract	6	2 (33%)	1 (17%)	1 (17%)	2 (33%)
Foveal hypoplasia	4	1 (25%)	1 (25%)	0	2 (50%)
**Laterality**					
Bilateral	71	16 (23%)	7 (10%)	5 (7%)	43 (60%)
Unilateral	29	2 (7%)	2 (7%)	1 (3%)	24 (83%)
**Systemic involvement**					
Isolated	45	10 (24%)	4 (9%)	1 (2%)	30 (67%)
Syndromic	55	8 (15%)	5 (9%)	5 (9%)	37 (67%)
**Family history of ocular malformation**					
Yes	18	6 (33%)	1 (6%)	2 (11%)	9 (50%)
No	82	13 (16%)	8 (10%)	4 (5%)	58 (71%)
**Previous genetic analyses**					
No	22	2 (9%)	2 (9%)	1 (5%)	17 (77%)
CGH-array	11	3 (27%)	1 (9%)	2 (18%)	5 (45%)
NGS	47	10 (21%)	4 (9%)	1 (2%)	32 (68%)
CGH-array + NGS	20	3 (15%)	2 (10%)	2 (10%)	13 (65%)
**WGS analysis configuration**					
Solo	1	0	0	0	1 (100%)
proband and one unaffected parent	2	0	1 (50%)	0	1 (50%)
proband and both unaffected parents	81	15 (19%)	7 (9%)	4 (37%)	56 (39%)
Other	16	4 (25%)	1 (6%)	2 (13%)	9 (56%)

Other: Genome analyses involving configurations other than solo or classic duo/trio (index case and asymptomatic parent[s]), including, for example, cases where two symptomatic individuals were jointly analyzed. Conclusive: Identification of a genotype considered causal or likely causal for the structural eye disease. VUS: Variant of Uncertain Significance. Partially conclusive: Identification of a genotype that accounts for some syndromic features but is not considered causal for the ocular phenotype. Inconclusive: No causal genotype identified.

WGS enabled a genetic diagnosis related to structural eye disease in 18 individuals (Table 2). The majority of diagnostic variants were small variants (SNV, indel), accounting for 13 of the 18 resolved cases. The remaining five cases involved SVs: three copy number variations (CNVs) and two balanced inversions. 14 identified variants (Table 3, pedigrees available in Supplementary Data 3) affected genes previously associated with structural eye disease [3, 5, 8, 10, 41, 42]: *ADAMTSL4, CHD7, CRYBA4, CRYBB1*, *CYP1B1, FOXC1*, *GJA8, HCCS, NHS, PAX6, PRSS56, PITX2* and *SLC38A8*. Additional diagnoses involved pathogenic variants in OMIM-listed genes associated with syndromic neurodevelopmental disorders where ocular anomalies have occasionally been reported, such as *SMARCA4* [43] and *WFS1* [44]. Furthermore, variants were identified in recently implicated genes in ocular malformations, including *ARHGAP35* [39] and *NR6A1* [45].

**Table 3:** Variant details from solved families and VUS.

Variant	Gene	Zygosity	Variant type	Inheritance	Variant known	frequency in gnomAD v4.1.0 (%)	Pathogenicity (ACMG criteria)	Causal of ocular anomaly ?	Likely visible using targeted DO187 panel	Individual ID	Phenotype
NM_012186.3:c.720 C > A p.(Cys240*) NC_000001.11:g.47417035 C > A	*FOXE3*	Het	SNV	Affected father	Chassaing et al. [36]	0.0017	P (PVS1, PS1, PM2)	No	+	I100	Axenfeld Rieger, microdontia
NM_005267.5:c.137 G > A p.(Gly46Glu) NC_000001.11:g.147908092 G > A	*GJA8*	Mos 20%	SNV	De novo	ClinVar 2103996	NA	P (PS2, PM1, PM2, PP3)	Yes	+/-	I081	Microphtalmia with congenital cataract, persistent hyperplastic anterior primary vitreous, pendular nystagmus
NM_019032.6:c.767_786del p.(Gln256Profs*38) NC_000001.11:g.150553758_150553777del	*ADAMTSL4*	Homo	Indel	Biparental	Knight et al. [68]	0.176	P (PVS1, PP5_verystrong)	Yes	-	I056	Bilateral Rieger anomaly, ectopia pupillae, ectopia lentis, posterior embryotoxon, microspherophakia, myopia
NM_000104.4:c.1064_1076del (p.Arg355Hisfs*69) NC_000002.12:g.38071278_38071290del	*CYP1B1*	Het	Indel	Unaffected father	ClinVar 282564	0.02	P (PVS1, PM2, PM3)	Yes	+	I049	Congenital glaucoma
NM_000104.4:c.182 G > A p.(Gly61Glu) NC_000002.12:g.38075207 C > T	*CYP1B1*	Het	SNV	Unaffected mother	Bejjani et al. [62]	0.031	P (PS1, PM1, PM2, PM3, PP3)	Yes	+	I049	Congenital glaucoma
NM_001395002.1:c.298 C > T p.(Gln100*) NC_000002.12:g.101824045 C > T	*MAP4K4*	Het	SNV	De novo	NA	NA	LP (PVS1, PS2, PM2)	Unlikely	-	I078	Coloboma (iris, choriortinal)
NM_020989.4:c.419 G > C p.(Arg140Pro) NC_000002.12:g.208128309 C > G	*CRYGC*	Het	SNV	Affected mother	NA	NA	VUS (PM1, PM2, PP3)	Unknown	+	I058	Congenital cataract, microphthalmia, nystagmus, myoisis
NM_001195129.2:c.69 C > A p.(Tyr23*) NC_000002.12:g.232520667 C > A	*PRSS56*	Homo	SNV	Biparental (consanguinity)	NA	NA	P (PVS1, PM2, PP4)	Yes	+	I088	Nanophthalmia
NM_032383.5:c.2995 C > T p.(Arg999*) NC_000003.12:g.149172202 C > T	*HPS3*	Het	SNV	Unaffected mother	NA	0.0004	LP (PVS1, PM2)	No	-	I051	Nystagmus, foveal hypoplasia, hypopigmentation of the fundus, recurrent pneumonia
NM_003106.4:c.115 G > T p.(Asp39Tyr) NC_000003.12:g.181712475 G > T	*SOX2*	Het	SNV	No found in unfacted mother	NA	NA	VUS (PM2, PP3)	Unknown	+	I052	Microphthalmia
NM_006005.3:c.1243_1245del p.(Val415del) NC_000004.12:g.6301038_6301040del	*WFS1*	Het	Indel	Affected mother	ClinVar 215406 Krutish et al. [51]: c.1243 G > T p.Val415Phe	0.007	P (PP1, PM2, PP4, PP5)	Likely	-	I027	Congenital cataract
NM_000325.6:c.350del p.(Pro117Argfs*91) NC_000004.12:g.110621227del	*PITX2*	Het	Indel	De novo	NA	NA	P (PVS1, PS2, PM2)	Yes	+	I063	Axenfeld Rieger, mandibular prognathia, ptosis, abnormal umbilicus morphology, abnormality of the dentition
NM_005245.4:c.13138+1 G > A p.? NC_000004.12:g.186595688 C > T	*FAT1*	Het	SNV	NA	NA	NA	LP (PVS1, PM2)	No	+	I093	Microphthalmia, cataract, glaucoma, microcephaly
seq[GRCh38]inv(6)(p25.1p25.3)dn NC_000006.12:g.(1713354_4870965)inv	Chr6 *(FOXC1)*	Het	SV	De novo	Individual previously reported in Plaisancié et al. [37]	NA	LP (de novo, transcript analysis show reduction of FOXC1 expression)	Yes	-	I030	Bilateral sclerocornea, left microphthalmia, mild DD, cavum septum pellucidum, microtia, hypertelorism, turricephaly, slender build, limited elbow extension, pes planus
NM_017780.4:c.4186-2 A > G p.? NC_000008.11:g.60837666 A > G	*CHD7*	Het	SNV	De novo	ClinVar 3601838	NA	P (PVS1, PS2, PM2)	Yes	+	I075	Bilateral coloboma (chorioretinal), right microphthalmia and microcornea
NM_014290.3:c.2159 G > A p.(Arg720Gln) NC_000009.12:g.97475462 G > A	*TDRD7*	Homo	SNV	Biparental (consanguinity)	NA	0.0002	VUS (PM2,PP3, BP1)	Unknown	-	I034	Bilateral cataract
NM_033334.4:c.844_851dup p.(Phe284Leufs*40) NC_000009.12:g.124536106_124536113dup	*NR6A1*	Het	Indel	Affected mother	NA	NA	LP (PVS1, PM2)	Likely	-	I009	Unilateral colobomatous microphthalmia
NM_000314.8:c.2 T > G p.? NC_000010.11:g.87864471 T > G	*PTEN*	Het	SNV	De novo	ClinVar 2674279	NA	P (PVS1, PS2, PM2, PP5_moderate)	Unlikely	-	I010	Bilateral microphthalmia, sclerocornea, optic nerve hypoplasia, DD, macrocephaly ( + 3 SD), enlarged sylvian cistern, ureteral duplication
seq[GRCh38]inv(10)(q24.3q26.1)dn NC_000010.11:g.(102253109_123049929)inv	Chr10 (PITX3)	Het	SV	De novo	NA	NA	VUS (de novo, predicted decrease *PITX3* expression)	Unknown	-	I005	Bilateral microphthalmia, severe global developmental delay, extrapyramidal dyskinesia
seq[GRCh38]inv(11)(p11.2p13) NC_000011.10:g.(31780037_44652946)inv	Chr11 *(PAX6)*	Het	SV	NA	NA	NA	P (PM2, PP1_strong, PP3, PP4)	Yes	-	I020	Bilateral aniridia
NM_015313.3:c.2614-7 G > A p.? NC_000011.10:g.120465230 G > A	*ARHGEF12*	Het	SNV	De novo	GWAS association of *ARHGEF12* with glaucoma Verma et al. [62]	NA	VUS (PS2, PM2, PM3) gene not OMIM morbid	Unknown	-	I031	Unilateral irido-chorio-retinal coloboma, sacral dimple
NM_015335.5:c.3484 T > C p.(Cys1162Arg) NC_000012.12:g.115991470 A > G	*MED13L*	Het	SNV	De novo	NA	NA	LP (PM1, PM2, PP2, PP3)	Unlikely	-	I061	Microphthalmia, Peters’ anomaly, DD, seizure, microcephaly, CFD, growth delay, congenital hip dislocation, bilateral talipes equinovarus, scoliosis
NM_021954.4:c.161 G > A p.(Cys54Tyr) NC_000013.11:g.20143128 C > T	*GJA3*	Het	SNV	Unaffected father	NA	NA	VUS (PM1, PM2, PP3, BS2)	Unknown	+	I054	Cataract, foveolar hypoplasia. Allergic eczema
NM_000275.3:c.1327 G > A p.Val443Ile NC_000015.10:g.27985101 C > T	*OCA2*	Het	SNV	Unaffected mother	Michaud et al. [65]	0.591	P (PS1, PM1, PM2, PP3, PP4)	No	-	I057	Nystagmus, foveal hypoplasia, hypopigmentation of the fundus, Hypopigmentation of the skin and the hair
seq[GRCh38]del(15)(q13.1q13.2)pat NC_000015.10:g.28100001_30040000del	Chr15	Het	CNV	Unaffected father	NA	NA	VUS (1 A, 2H, 3 A, 5 A, score 0.6)	Unknown	-	I053	Bilateral colobomatous microphthalmia, autism spectrum disorder, developmental delay
NM_001080442.3:c.932 C > T p.(Pro311Leu) NC_000016.10:g.84017161 G > A	*SLC38A8*	Comp Het	SNV	Unaffected father	NA	0.0006	LP (PM1, PM2, PP1, PP3, PP4)	Yes	+	I047	Foveal hypoplasia
NM_001080442.3:c.682 G > A p.(Gly228Arg) NC_000016.10:g.84029502 C > T	*SLC38A8*	Comp Het	SNV	Unaffected mother	Kuht et al. [7] VUS	0.003	LP (PM1, PM2, PP1, PP3, PP4)	Yes	+	I047	Foveal hypoplasia
seq[GRCh38]dup(17)(q12q21.2)dn NC_000017.11:g.(39600001_40300000)dup	Chr17	Het	CNV	De novo	NA	NA	VUS (1 A, 2B, 3 A, 5 A, score 0.15)	Unknown	-	I064	Unilateral anophthalmia (therapeutic termination of the pregnancy)
NM_006852.6:c.1025 T > C p.(Leu342Ser) NC_000017.11:g.62573271 T > C	*TLK2*	Het	SNV	De novo	NA	NA	LP (PS2, PM2, PP3)	Unlikely	-	I080	Coloboma, DD, gastroesophageal reflux
NM_001353214.3:c.916 C > T p.(Gln306*) NC_000018.10:g.49286464 G > A	*DYM*	Homo	SNV	Biparental (consaguinity) Affected sister: Het	Akalin et al. [69]	NA	P (PVS1, PS1, PM2, PP4)	Unlikely	-	I087	Congenital cataract, DD, hypotonia, growth delay, pyramidal signs, microcephaly
NM_003072.5:c.1757_1760del p.(Lys586Argfs*26) NC_000019.10:g.10996376_10996379del	*SMARCA4*	Het	Indel	De novo	ClinVar 545889 & Individual previously reported in Chesneau et al. [38]	0.0001	P (PVS1, PS2, PM2, PP5)	Likely	-	I023	Unilateral microphthalmia, optic nerve hypoplasia, retinal dysplasia
NM_003072.5:c.1761+2 T > G p.? NC_000019.10:g.10996382 T > G	*SMARCA4*	Het	SNV	Unaffected mother	Individual previously reported in Chesneau et al. [38]	NA	LP (PVS1, PM2)	Likely	-	I062	Bilateral iris coloboma, expressive language delay, few cafe-au-lait spots, joint hypermobility, abnormal pinna morphology, long eyelashes, specific learning disability
NM_004491.5:c.1849C>T p.(Arg617*) NC_000019.10:g.46920524 C > T	*ARHGAP35*	Het	SNV	De novo	Individual previously reported in Reis et al. [38]	NA	P (PVS1, PS2, PM2)	Likely	-	I010	Bilateral microphthalmia, sclerocornea, optic nerve hypoplasia, DD, macrocephaly ( + 3 SD), enlarged sylvian cistern, ureteral duplication
NM_001887.4:c.576-637_587del seq[GRCh38]del(22)(q12.1)pat NC_000022.11:g.(26599662_26600310)del	*CRYBB1*	Het	CNV	Affected Father	NA	NA	P (1 A, 2E, 3A1, 5D, score 1.05)	Yes	+/-	I013	Bilateral colobomatous microphthalmia, ocular anterior segment dysgenesis, cataract, glaucoma
NM_001886.3:c.220 T > C p.(Tyr74His) NC_000022.11:g.26625542 T > C	*CRYBA4*	Het	SNV	De novo	NA	NA	P (PS2, PM1, PM2, PP3_strong)	Yes	-	I089	Bilateral congenital cataract. Sacral dimple
seq[GRCh38] del(X)(Xp22.2)mat NC_000023.11:g.(10900001_11120000)del	*ChrX* *(HCCS)*	Het	CNV	Affected mother	NA	NA	P (1 A, 2C-1, 3 A, 5D, score 1.35)	Yes	+	I014	Right: complex microphthalmia with microcornea, left: iris hypoplasia
seq[GRCh38] del(X)(Xp22.2p22.13)pat NC_000023.11:g.16897001_17560000del	*ChrX* *(NHS)*	Het	CNV	Affected father	NA	NA	LP (1 A, 2C-1, 3 A, score 0,9)	Partly	+	I093	Microphthalmia, cataract, glaucoma, microcephaly
seq[GRCh38] del(X)(Xp22.13)dn NC_000023.11:g.(17649001_17737000)del	*ChrX* *(NHS)*	Hemi	CNV	De novo	NA	NA	P (1 A, 2D-4, 3 A, 5 A, score 1.35)	Yes	+	I028	Microphtalmia with congenital cataract and glaucoma
NM_203475.3:c.1289 A > G p.(Tyr430Cys) NC_000023.11:g.48520379 A > G	*PORCN*	Hemi	SNV	Unaffected mother	NA	NA	VUS (PM1, PM2, PP3)	Unknown	+	I015	Microphthalmia, arachnoid cyst, hydronephrosis, hypothyroidism, growth delay, CFD, bowed femur
NM_001110792.2:c.799 C > T p.(Arg267*) NC_000023.11:g.154031065 G > A	*MECP2*	Het	SNV	De novo	Yusufzai et al. [56]	NA	P (PS2, PM2, PP3, PP5).	Unlikely	-	I060	Bilateral irido-chorio-retinal coloboma, global developmental delay, abnormal eating behavior

Variants were classified according to ACMG criteria [60,61]. Genotype causality regarding the ocular anomaly was assessed based on current literature data and categorized as follows: ‘no’ (not causative), ‘unlikely’ (unlikely to be causative), ‘unknown’ (insufficient data), ‘likely’ (likely to be causative), or ‘yes’ (confirmed causative). *CNV* Copy Number Variant, *Comp* Compound Heterozygote, *GWAS* Genome Wide Association Study, Het Heterozygote, *Homo* Homozygote, *LP* Likely Pathogenic, *NA* Not Available, *P* Pathogenic, *SNV* Single Nucleotide Variant, *VUS* Variant of Uncertain Significance.

A female patient (I093) presenting with severe ocular phenotype (bilateral complex microphthalmia with cataract and glaucoma) was found to carry a familial partial deletion of *NHS* at heterozygous state. While this deletion may contribute to the phenotype, the severity of the ocular manifestations exceeds what is typically observed in female heterozygous carriers of *NHS* variants, who usually present with cataracts showing incomplete penetrance [46].

In six individuals with ocular malformations in a syndromic context, we identified a (likely) pathogenic variant that partially explain the clinical presentation but are unlikely to account fully for the ocular phenotype. These variants were located in genes associated with neurodevelopmental disorders in which structural eye anomalies have not yet been reported (Table 3, pedigrees available in Supplementary Data 3): *DYM* [47], *MAP4K4* [48], *MECP2* [49], *MED13L* [50], *PTEN* [51], *TLK2* [52]. A dual-etiology mechanism may be at play in several of these cases. Notably, in the individual (I010) carrying the *PTEN* variant, likely responsible for the DD and macrocephaly, a second variant in *ARHGAP35*, likely responsible for the ocular phenotype, was also identified. Individual I087 carries a pathogenic homozygous variant in *DYM* associated with Dyggve-Melchior-Clausen disease, explaining the extra-ocular features. However, this does not explain the familial congenital cataracts (observed in the proband, mother, and sister), as the relatives are only heterozygous for this variant and present with an isolated ocular phenotype.

In eight individuals, we identified variants of uncertain significance (VUSs) that do not meet the ACMG criteria to be classified as likely pathogenic [53, 54]. Six of these VUSs were located in genes previously implicated in eye development anomalies [3, 8] (Table 3, pedigrees available in Supplementary Data 3): *CRYGC*, *GJA3*, *PITX3*, *PORCN*, *SOX2* and *TDRD7*. One variant was found in *ARHGEF12*, a gene not previously associated with structural eye defects in the literature, but which is expressed during ocular development and has been linked to an increased risk of glaucoma [55]. In addition, we detected two CNVs that do not encompass any genes currently known to be involved in eye development.

Finally, four cases had single heterozygous recessive alleles identified with no second variant detected (Table 3, pedigrees available in Supplementary Data 3): *FAT1* [56], *FOXE3* [57], *OCA2* [58] and *HPS3* [7]. In the case of *FOXE3* [57], loss-of-function variants in this gene, such as the one identified here, are more commonly associated with recessive inheritance.

Of note, an incidental finding has been found in 5 individuals with pathogenic variants in genes associated with cancer predisposition (*BRCA2*: 2, *BRCA1*: 1, *PALB2*: 1) or in susceptibility to malignant hyperthermia (*RYR1*: 1).

## Discussion

### Diagnostic yield

Here, we report a large cohort of 100 individuals with structural eye disease genetically explored using short-read WGS. This analysis provided 18 diagnoses regarding the ocular phenotype. This result is lower than the diagnostic yield observed in other cohorts of individuals with structural eye anomalies analysed using short-read WGS (24-77%) (Table 1) [21, 22, 27]. This could be explained by the fact that WGS was used as a second-line analysis after targeted NGS and/or CGH-array for three quarters (78%) of the patients in our cohort. However, we observed a similar diagnostic yield (15%) when considering only individuals without prior NGS analysis (*n* = 33).

Patients with a more severe ocular defect appear more likely to receive a molecular diagnosis, for example when the defect is bilateral (23% vs. 7%) or complex (M/A + ASD: 22% vs. isolated M/A: 13%). These findings are consistent with previous studies suggesting that the likelihood of identifying a genetic cause increases with the severity of the ocular malformation [3, 23, 36].

In addition, the nature of ocular defect also modifies the mutation detection rate. The group of ocular malformations with the lowest diagnostic yield are isolated ocular colobomas, which confirms the data we previously found in targeted NGS analysis [59]. In their cohort, Jackson et al. [21] reported a higher diagnostic yield in individuals with congenital cataracts (45%) compared to those with M/A (16%) or ASD (13%). Similarly, in a cohort of 52 patients with congenital cataracts studied by Ma et al. [27], 14 remained undiagnosed after the CGH-array and WES/targeted NGS panel approaches were employed. Of these, five were resolved through the additional diagnostic yield of WGS (5/14, 36%).

Moreover, diagnostic yields were similar between patients with isolated ocular involvement (22%) and those with associated systemic features (15%), consistent with previously reported data in the literature [22, 23]. While Neuhann et al. [23] reported higher yields in individuals with anterior segment dysgenesis (ASD) and extra-ocular features, yields for microphthalmia/anophthalmia (M/A) and coloboma remained similar regardless of systemic involvement. Our results showed no significant disparity between isolated and syndromic phenotypes across ocular subtypes, except for M/A + ASD: ASD (18% [2/11] isolated vs. 22% [2/9] syndromic), M/A and coloboma (11% [2/19] vs. 13% [3/24]), and M/A + ASD (33% [4/12] vs. 13% [2/15]). Notably, in six patients with extra-ocular features, the identified variants accounted for the systemic manifestations but failed to explain the ocular phenotype (“Partially conclusive” cases).

### Gain in comparison with other existing technics

When considering the gain of WGS compared to targeted NGS panels, at least half of the diagnoses (*n* = 10/18) would have been missed using our 187 genes NGS panel (list of genes in Supplementary Data 1). A WES approach would likely have identified 16 of these 18 diagnoses (as it could not detect the two balanced inversions). This contrast with other cohorts in which the variants reported are SNVs in the coding regions of genes that are already associated with ocular malformations, which would likely have been identified using a targeted NGS panel. Jackson et al. [21] reported on a cohort of 293 patients with non-retinal developmental eye disorders in the United Kingdom who underwent WGS, achieving an overall diagnosis rate of 24.9%. All of the reported variants were located within or close to coding regions. The authors discuss the fact that their analysis of non-coding variants was limited by the UK Genomics England 100,000 Genomes Project pipeline to splice regions only. They also did not report any SV. Therefore, if we focus on individuals with either M/A, ASD or cataracts, most of the variants found in their cohort would likely have been identified using our targeted NGS panel (only 3/37 variants, 8%, were located in genes absent from our panel).

As previously shown [60], short-read WGS demonstrates an advantage in the identification of balanced and unbalanced SVs in comparison to NGS panel, WES and CGH-array. We were able to detect two inversions and three CNVs. The two inversions are located close to genes known for their implication in eye development (10 kb 3’ from *PAX6* and 100 kb 5’ from *FOXC1* respectively) and are predicted to disrupt their expression regulation by separating these genes from their cis regulatory elements [26, 37]. In addition to the two inversions, WGS allows easier detection of CNVs such as the intragenic 649 bp deletion of *CRYBB1* that delete only 11 bases of exon 6 making it difficult to detect using targeted NGS usually covering only exons and exon-introns boundaries.

Although gene panels are regularly updated, they remain inherently limited and cannot be fully exhaustive. In contrast, WGS (and WES) enables the identification of variants in genes not typically included in eye malformations panels, such as *ADAMTSL4* (commonly associated with ectopia lentis) [61] or *WFS1* (typically linked to optic atrophy) [44]. WGS also facilitated the detection of variants in newly implicated genes, such as *NR6A1* [45]. In addition, it enabled the identification of novel candidate genes, such as *ARHGAP35*, in which we detected a de novo nonsense variant in an individual with bilateral complex syndromic microphthalmia. Collaboration with other research teams subsequently confirmed the involvement of *ARHGAP35* in human eye morphogenesis [40]. Furthermore, unlike targeted NGS panels, WGS offers the advantage of streamlined reanalysis, allowing the detection of variants in genes discovered after the initial sequencing.

Pangenomic sequencing also facilitates the exploration of genes that are not usually included in a targeted NGS eye panel, as they are associated with human diseases in which eye malformation is not a common feature. For instance, we found truncating rare variant in *SMARCA4* in two unrelated individuals (previously reported) [38]. This gene has been associated with two distinct phenotypes in the literature: Coffin-Siris syndrome and rhabdoid tumour predisposition [43]. While these conditions are not classically linked to eye development anomalies, cases of M/A have been occasionally reported in individuals with a loss of function variant in *SMARCA4* [43].

However, in some patients, the identified (likely) pathogenic variants are unlikely to account for the ocular phenotype, as they were located in genes not currently associated with eye malformations, including *DYM* [62]*, MAP4K4* [48]*, MECP2* [49], *MED13L* [50], *PTEN* [51] and *TLK2* [52]. While these findings partially addressed the clinical concerns, they do not provide a complete diagnosis based on current knowledge. Notably, a second variant, likely causative of the ocular phenotype, was identified in *ARHGAP35* in the individual carrying the *PTEN* variant associated with Cowden syndrome (I010). Such instances of multiple molecular diagnoses are relatively frequent, occurring in approximately 3.5% of individuals in intellectual disability cohorts [63].

### Interpretation difficulties

While the diagnostic utility of WGS for individuals with structural eye diseases is undeniable, this approach also presents new challenges. The most important of these might be its difficulty of interpretation. The millions of variants detected by WGS necessitate robust bioinformatics pipelines for variant calling, annotation and filtering. Prior to the advent of WGS, clinical genetic testing was largely confined to coding regions. It is now well described that variants in non-coding regions (including intergenic regions, enhancers, promoters, introns, 5’ and 3’ untranslated regions (UTRs)) can contribute to human diseases [26, 64, 65]. However, interpreting these variants remains particularly challenging and depends on the development of reliable databases and predictive tools. One such example is MORFEEdb (Mutation on Open Reading FramE annotation), a tool recently developed to enhance the annotation of SNVs within human 5′ UTRs [66]. As a result, some potentially pathogenic variants may have been inadvertently filtered out by our pipeline due to current limitations in knowledge or annotation capabilities. This underscores the importance of regular genome reanalysis to capture newly recognized disease mechanisms. Finally, trio-based analysis (involving the proband and both parents) plays a key role in variant interpretation. This approach was favored in the France Genomic Medicine 2025 initiative [16], from which this cohort originates, and has demonstrated a diagnostic advantage over solo sequencing, with diagnostic yields of 29.1% and 20.7%, respectively.

In line with the challenges of variant interpretation, we identified VUS in nine patients (Table 3). These variants represent potential candidates for explaining the observed ocular phenotypes and may contribute to the discovery of new genes or molecular mechanisms. However, additional analyses are required to confirm their pathogenicity. Five of the identified VUS are SNVs affecting the coding sequence of genes previously associated with genetic eye diseases: *CRYGC*, *GJA3*, *PORCN*, *SOX2* and *TDRD7* [3, 8]. One case involved a de novo 20 Mb inversion on chromosome 10, with one breakpoint located 10 kb upstream of the 5′ UTR of the *PITX3* gene. The patient presented with bilateral microphthalmia and severe DD, a phenotype more severe than typically reported for *PITX3*-related disorders, which are usually limited to ASD without DD [67]. This discrepancy may be due to ectopic *PITX3* expression, disruption of an additional gene by the inversion or the presence of another undetected pathogenic variant. In another case, we identified a de novo intronic variant in *ARHGEF12*, predicted to affect splicing. Although this gene is not currently associated with a Mendelian disorder, it is expressed during ocular development, is intolerant to loss-of-function variants and has been implicated in glaucoma susceptibility through genome-wide association studies [55]. The final two VUS consist of two CNVs (del 15q13.1q13.2 and dup 17q12q21.2) for which current evidence is insufficient to establish pathogenicity.

In four patients, we identified heterozygous (likely) pathogenic variants in genes typically associated with autosomal recessive inheritance: *FAT1* [56], *FOXE3* [57], *HPS3* [68], *OCA2* [58]. Despite extensive investigation, no second pathogenic variant was found in these genes. While the patients’ phenotypes were broadly consistent with those described for these genes, it remains unclear whether the identified heterozygous variant contributes to the phenotype alongside an undetected second variant or whether it represents an incidental finding, with the phenotype instead explained by a variant in another gene.

### Incidental findings

In addition, genomic exploration exposes to the discovery of incidental findings. In our cohort, we found an incidental finding in 5% of patients, which is consistent with the literature, highlighting the relative frequency of such findings [69]. Notably, these findings were not actively sought but emerged unintentionally during the search for the primary genetic diagnosis. This raise ethical questions and can be challenging for both patients and clinicians who are dealing with this type of results. The pathogenic variants were located in actionable genes according to the American College of Medical Genetics and Genomics (*BRCA2*: 2, *BRCA1*: 1, *PALB2*: 1, *RYR1*: 1) [70].

### Limits of short-read WGS

Following analysis, two-thirds of patients remained without a molecular diagnosis or a variant of interest. Beyond the current limitations in knowledge, short-read WGS also has inherent technical constraints. The commonly used human reference genome (GRCh38) contains unresolved regions, particularly in large repetitive arrays, as well as pericentromeric and subtelomeric regions, which lead to alignment difficulties for short reads. Similarly, the detection of complex SVs remains challenging, especially when breakpoints lie within regions poorly covered or inaccessible to short-read sequencing technologies. Long-read sequencing (LRS), which enables sequencing of reads spanning tens to hundreds of kilobases, offers the potential to overcome these limitations [71]. The adoption of LRS is expected to improve diagnostic yield in the coming years. For example, LR-WGS has recently allowed a better understanding of the molecular mechanism behind M/A spectrum linked to complex rearrangement in 13q region, by identifying a complex SV in this region [72]. However, the added value of LRS in the context of structural eye diseases remains to be fully determined.

A remaining issue with WGS is its cost compared to CGH-array and targeted NGS analysis. Although the financial and technical cost of WGS has fallen sharply in recent years, it remains more expensive that first-line analysis. Moreover, it generates much more data that then needs to be stored, while data storage is now well known to have a huge environmental impact [73].

## Conclusion

To conclude, we observed an 18% diagnostic yield using WGS in our cohort of 100 patients with ocular malformations. Although a large number of individuals remain without a molecular diagnosis, considering thediagnostic contribution of WGS, this analysis should be considered and standardized for patients with ocular malformations. The arrival of LRS should further increase the number of molecular diagnoses made, pointing to a promising future in patient care.

## Supplementary information

Supplementary Data 1: List of genes analyzed in the three NGS ocular gene panel (DO24, DO119 and DO187).

Supplementary data 3: pedigrees of families with a reported variant

Supplementary Data 2: Comprehensive phenotypic and genotypic profiles of included individuals, including family history and prior genetic investigations.

Supplementary data 4: Full list of AURAGEN CONSORTIUM members

## Data Availability

The molecular datasets presented in this study can be found in online repositories on the website: https://pfmg2025.fr/. All reported variant have been reported in Clinvar database (SCV007495238).
