# Supplementary material for: Unveiling ocular developmental disorders through short-read whole-genome sequencing
Source: Eur J Hum Genet. 2026 Jul 14;34(10):1573–84. doi: 10.1038/s41431-026-02160-4 (PMC13634046; doi:10.1038/s41431-026-02160-4)

Supplementary data 3: pedigrees of families with a reported variant

WT: wild type

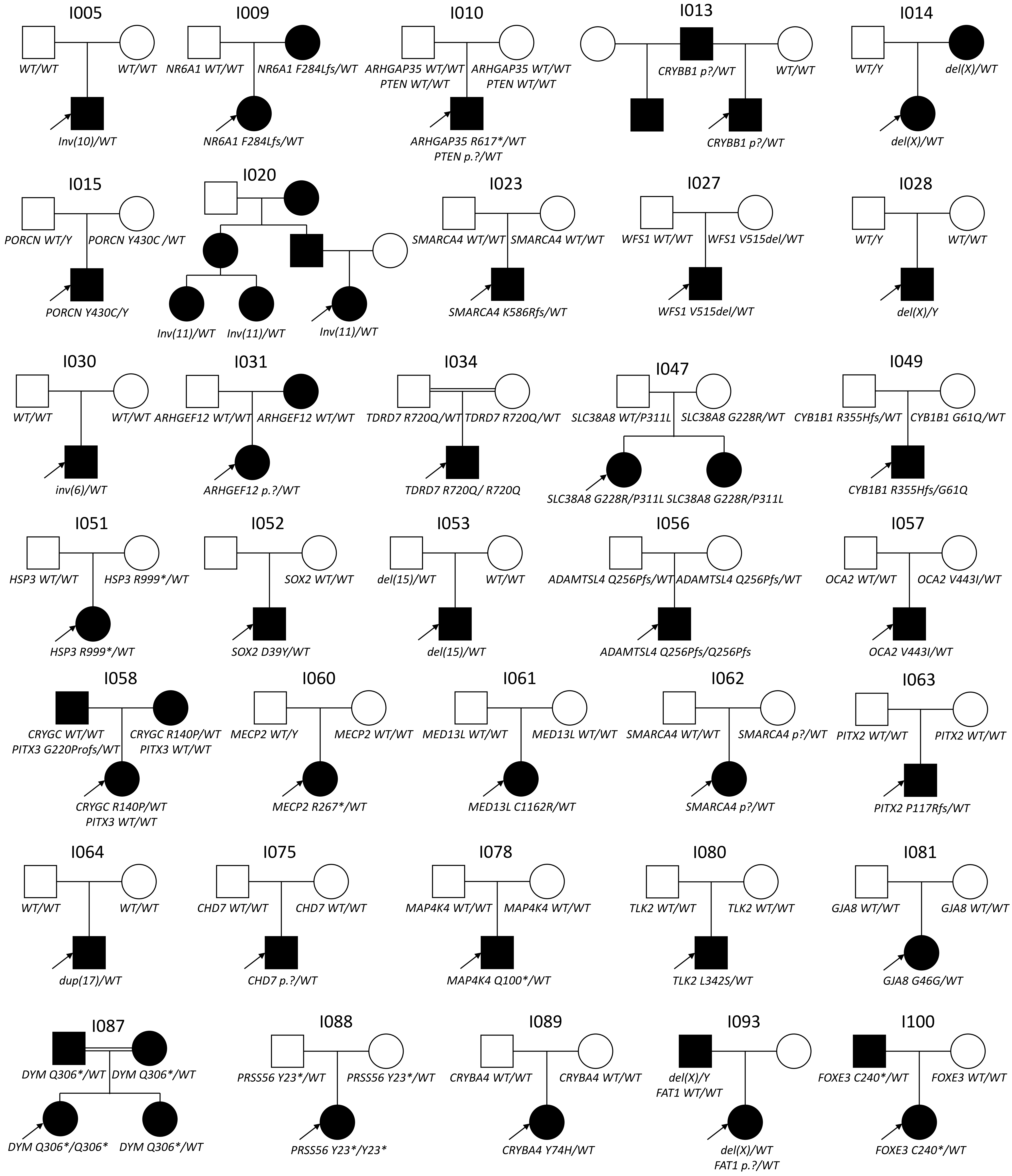

Supplement: Supplementary file 1 [file 41431_2026_2160_MOESM2_ESM.pdf]
