# Supplementary material for: Unveiling ocular developmental disorders through short-read whole-genome sequencing
Source: Eur J Hum Genet. 2026 Jul 14;34(10):1573–84. doi: 10.1038/s41431-026-02160-4 (PMC13634046; doi:10.1038/s41431-026-02160-4)
Supplement: Supplementary file 2 [file 41431_2026_2160_MOESM1_ESM.docx]

**Supplementary Data 1 :** List of genes analyzed in the three NGS ocular gene panel (DO24, DO119 and DO187).

| **Gene** | **RefSeq** | **DO24** | **DO119** | **DO187** |
| --- | --- | --- | --- | --- |
| ACTB | NM_001101.3 |  | X | X |
| ACTG1 | NM_001199954.1 |  | X | X |
| ADAMTS10 | NM_030957.4 |  |  | X |
| ADAMTS17 | NM_139057.4 |  |  | X |
| ADAMTS18 | NM_199355.3 |  | X | X |
| ADAMTS9 | NM_182920.2 |  |  | X |
| ADNP | NM_001282531.1 |  | X | X |
| AHR | NM_001621.4 |  | X | X |
| ALDH1A3 | NM_000693.3 |  | X | X |
| ALX1 | NM_006982.3 |  |  | X |
| ANGPT1 | NM_001146.5 |  |  | X |
| ANK3 | NM_020987.5 |  |  | X |
| ATOH7 | NM_145178.3 |  | X | X |
| B3GLCT | NM_194318.3 | X | X | X |
| BCOR | NM_001123385.1 | X | X | X |
| BEST1 | NM_001139443.1 |  | X | X |
| BFSP1 | NM_001195.4 |  | X | X |
| BFSP2 | NM_003571.3 |  | X | X |
| BHLHE40 | NM_003670.2 |  | X | X |
| BMP4 | NM_001202.4 |  | X | X |
| BMP7 | NM_001719.2 |  | X | X |
| BMPR1A | NM_004329.3 |  |  | X |
| BMPR1B | NM_001203.3 |  |  | X |
| BRPF1 | NM_001003694.2 |  |  | X |
| C12orf57 | NM_138425.3 | X | X | X |
| CAPN15 | NM_005632.3 |  |  | X |
| CDH2 | NM_001792.5 |  |  | X |
| CDH4 | NM_001794.5 |  |  | X |
| CDON | NM_001378964.1 |  |  | X |
| CELF1 | NM_001376376.1 |  |  | X |
| CENPF | NM_016343.4 |  |  | X |
| CHD7 | NM_017780.3 |  | X | X |
| CHMP4B | NM_176812.4 |  | X | X |
| CHRDL1 | NM_001143981.2 |  |  | X |
| CNBP | NM_001127193.1 |  |  | X |
| COL4A1 | NM_001845.5 |  | X | X |
| COL6A3 | NM_057164.4 |  |  | X |
| COX7B | NM_001866.2 |  | X | X |
| CPAMD8 | NM_015692.2 |  | X | X |
| CREBBP | NM_004380.2 |  | X | X |
| CRIM1 | NM_016441.3 |  |  | X |
| CRYAA | NM_000394.4 |  |  | X |
| CRYAB | NM_001289808.1 |  | X | X |
| CRYBA1 | NM_005208.4 |  | X | X |
| CRYBB1 | NM_001887.3 |  | X | X |
| CRYBB2 | NM_000496.2 |  | X | X |
| CRYBB3 | NM_004076.4 |  | X | X |
| CRYGC | NM_020989.4 |  |  | X |
| CRYGD | NM_006891.3 |  | X | X |
| CTNNB1 | NM_001904.4 |  |  | X |
| CYP1B1 | NM_000104.3 | X | X | X |
| CYP26A1 | NM_000783.4 |  |  | X |
| CYP27A1 | NM_000784.4 |  |  | X |
| DBN1 | NM_001364151.2 |  |  | X |
| EDNRB | NM_000115.3 |  | X | X |
| EP300 | NM_001429.3 |  | X | X |
| EPHA2 | NM_004431.4 |  | X | X |
| EYA1 | NM_000503.5 |  | X | X |
| FAT1 | NM_005245.3 |  | X | X |
| FBXW11 | NM_012300.2 |  |  | X |
| FNBP4 | NM_001318339.1 |  | X | X |
| FOXC1 | NM_001453.2 | X | X | X |
| FOXC2 | NM_005251.3 |  |  | X |
| FOXE3 | NM_012186.2 | X | X | X |
| FOXL2 | NM_023067.4 |  |  | X |
| FRAS1 | NM_025074.6 |  | X | X |
| FREM1 | NM_144966.5 |  | X | X |
| FRMD7 | NM_194277.3 |  |  | X |
| FRS2 | NM_001278351.1 |  |  | X |
| FZD4 | NM_012193.3 |  | X | X |
| FZD5 | NM_003468.3 |  | X | X |
| GALT | NM_000155.4 |  |  | X |
| GDF3 | NM_020634.1 |  | X | X |
| GDF6 | NM_001001557.2 |  | X | X |
| GJA1 | NM_000165.5 |  |  | X |
| GJA8 | NM_005267.4 |  | X | X |
| HCCS | NM_005333.4 |  | X | X |
| HESX1 | NM_003865.2 |  | X | X |
| HMGB3 | NM_001301231.1 |  | X | X |
| HMX1 | NM_018942.2 |  | X | X |
| HSF4 | NM_001538.3 |  | X | X |
| IGBP1 | NM_001551.2 |  | X | X |
| IPO13 | NM_014652.3 |  | X | X |
| ITPR1 | NM_001099952.2 |  | X | X |
| KIF11 | NM_004523.4 |  |  | X |
| KIF17 | NM_001122819.3 |  |  | X |
| KIF26B | NM_018012.4 |  |  | X |
| LIM2 | NM_030657.3 |  | X | X |
| LONP1 | NM_004793.4 |  |  | X |
| LRP5 | NM_002335.3 |  | X | X |
| LTBP2 | NM_000428.2 |  | X | X |
| MAB21L1 | NM_005584.5 |  |  | X |
| MAB21L2 | NM_006439.4 | X | X | X |
| MAF | NM_005360.4 |  | X | X |
| MFRP | NM_031433.3 | X | X | X |
| MICU1 | NM_001195518.2 |  |  | X |
| MIP | NM_012064.3 |  | X | X |
| MITF | NM_198159.2 |  | X | X |
| MSX2 | NM_002449.4 |  | X | X |
| MYOC | NM_000261.2 |  |  | X |
| MYRF | NM_001127392.3 |  |  | X |
| NAA10 | NM_003491.3 |  | X | X |
| NCAPG2 | NM_017760.7 |  |  | X |
| NDP | NM_000266.3 |  | X | X |
| NDUFB11 | NM_001135998.3 |  |  | X |
| NHS | NM_001291867.2 |  |  | X |
| NUP188 | NM_015354.3 |  |  | X |
| OCLN | NM_001205254.2 |  |  | X |
| OCRL | NM_000276.3 |  | X | X |
| OTX2 | NM_021728.3 | X | X | X |
| PACS1 | NM_018026.4 |  |  | X |
| PAX2 | NM_003990.4 | X | X | X |
| PAX3 | NM_181457.3 |  | X | X |
| PAX6 | NM_001258462.1 | X | X | X |
| PDE6D | NM_002601.3 |  | X | X |
| PDGFRA | NM_006206.6 |  |  | X |
| PIGL | NM_004278.3 |  | X | X |
| PIK3R1 | NM_181523.3 |  |  | X |
| PITX2 | NM_001204397.1 | X | X | X |
| PITX3 | NM_005029.3 | X | X | X |
| PORCN | NM_203475.2 |  | X | X |
| PQBP1 | NM_005710.2 |  | X | X |
| PRR12 | NM_020719.3 |  |  | X |
| PRRX1 | NM_022716.4 |  |  | X |
| PRSS56 | NM_001195129.1 | X | X | X |
| PTCH1 | NM_000264.3 |  | X | X |
| PUF60 | NM_078480.3 |  |  | X |
| PXDN | NM_012293.2 | X | X | X |
| RAB18 | NM_021252.4 |  | X | X |
| RAB3GAP1 | NM_001172435.1 |  | X | X |
| RAB3GAP2 | NM_012414.3 |  | X | X |
| RARA | NM_000964.4 |  |  | X |
| RARB | NM_000965.4 | X | X | X |
| RAX | NM_013435.2 |  | X | X |
| RBM24 | NM_001143942.2 |  |  | X |
| RBP4 | NM_006744.3 |  | X | X |
| RERE | NM_012102.3 |  | X | X |
| SALL1 | NM_002968.2 |  | X | X |
| SALL2 | NM_005407.2 |  | X | X |
| SEMA3E | NM_012431.2 |  | X | X |
| SHH | NM_000193.3 |  | X | X |
| SIL1 | NM_001037633.1 |  | X | X |
| SIX3 | NM_005413.3 |  | X | X |
| SIX6 | NM_007374.2 | X | X | X |
| SLC16A12 | NM_213606.3 |  | X | X |
| SLC25A24 | NM_013386.4 |  | X | X |
| SLC38A8 | NM_001080442.3 |  |  | X |
| SLU7 | NM_006425.5 |  |  | X |
| SMCHD1 | NM_015295.2 |  | X | X |
| SMG9 | NM_019108.4 |  |  | X |
| SMO | NM_005631.4 |  | X | X |
| SMOC1 | NM_001034852.2 | X | X | X |
| SNX3 | NM_003795.6 |  |  | X |
| SOX10 | NM_006941.3 |  | X | X |
| SOX11 | NM_003108.4 |  |  | X |
| SOX14 | NM_004189.3 |  | X | X |
| SOX2 | NM_003106.3 | X | X | X |
| SOX21 | NM_007084.3 |  | X | X |
| SOX3 | NM_005634.3 |  |  | X |
| SOX4 | NM_003107.3 |  |  | X |
| SRD5A3 | NM_024592.4 |  | X | X |
| STRA6 | NM_001199042.1 | X | X | X |
| TBC1D20 | NM_144628.3 |  | X | X |
| TBX2 | NM_005994.4 |  |  | X |
| TEK | NM_000459.5 |  |  | X |
| TENM3 | NM_001080477.3 | X | X | X |
| TFAP2A | NM_003220.2 |  | X | X |
| TMEM98 | NM_015544.2 |  | X | X |
| TMX3 | NM_019022.3 |  | X | X |
| TRIM44 | NM_017583.5 |  | X | X |
| TSPAN12 | NM_012338.4 |  |  | X |
| VAX1 | NM_001112704.1 |  | X | X |
| VAX2 | NM_012476.2 |  | X | X |
| VCAN | NM_004385.5 |  |  | X |
| VIM | NM_003380.3 |  | X | X |
| VPS4A | NM_013245.3 |  |  | X |
| VRK2 | NM_001288837.2 |  |  | X |
| VSX2 | NM_182894.2 | X | X | X |
| WDR37 | NM_014023.4 |  |  | X |
| WNT2B | NM_024494.3 |  |  | X |
| WNT7B | NM_058238.2 |  |  | X |
| WT1 | NM_024426.4 |  | X | X |
| YAP1 | NM_001130145.2 |  | X | X |
| ZEB2 | NM_014795.3 |  | X | X |
| ZFHX4 | NM_024721.5 |  |  | X |
| ZIC5 | NM_033132.3 |  |  | X |
| ZNF408 | NM_024741.3 |  |  | X |
